# Submersible voltammetric sensing probe for rapid and extended remote monitoring of opioids in community water systems

**DOI:** 10.1007/s00604-024-06520-z

**Published:** 2024-07-12

**Authors:** Jiachi Zhou, Shichao Ding, Samar S. Sandhu, An-Yi Chang, Anubhap Taechamahaphan, Shipra Gudekar, Joseph Wang

**Affiliations:** Department of Nanoengineering, University of California San Diego, La Jolla, CA 92093 USA

**Keywords:** Opioids, Wastewater-based epidemiology, Submersible probes, Fentanyl, Remote sensing, Electrochemical sensors, Square wave voltammetry

## Abstract

The intensifying global opioid crisis, majorly attributed to fentanyl (FT) and its analogs, has necessitated the development of rapid and ultrasensitive remote/on-site FT sensing modalities. However, current approaches for tracking FT exposure through wastewater-based epidemiology (WBE) are unadaptable, time-consuming, and require trained professionals. Toward developing an extended in situ wastewater opioid monitoring system, we have developed a screen-printed electrochemical FT sensor and integrated it with a customized submersible remote sensing probe. The sensor composition and design have been optimized to address the challenges for extended in situ FT monitoring. Specifically, ZIF-8 metal–organic framework (MOF)-derived mesoporous carbon (MPC) nanoparticles (NPs) are incorporated in the screen-printed carbon electrode (SPCE) transducer to improve FT accumulation and its electrocatalytic oxidation. A rapid (10 s) and sensitive square wave voltammetric (SWV) FT detection down to 9.9 µgL^−1^ is thus achieved in aqueous buffer solution. A protective mixed-matrix membrane (MMM) has been optimized as the anti-fouling sensor coating to mitigate electrode passivation by FT oxidation products and enable long-term, intermittent FT monitoring. The unique MMM, comprising an insulating polyvinyl chloride (PVC) matrix and carboxyl-functionalized multi-walled carbon nanotubes (CNT-COOH) as semiconductive fillers, yielded highly stable FT sensor operation (> 95% normalized response) up to 10 h in domestic wastewater, and up to 4 h in untreated river water. This sensing platform enables wireless data acquisition on a smartphone via Bluetooth. Such effective remote operation of submersible opioid sensing probes could enable stricter surveillance of community water systems toward timely alerts, countermeasures, and legal enforcement.

**Graphical abstract:**

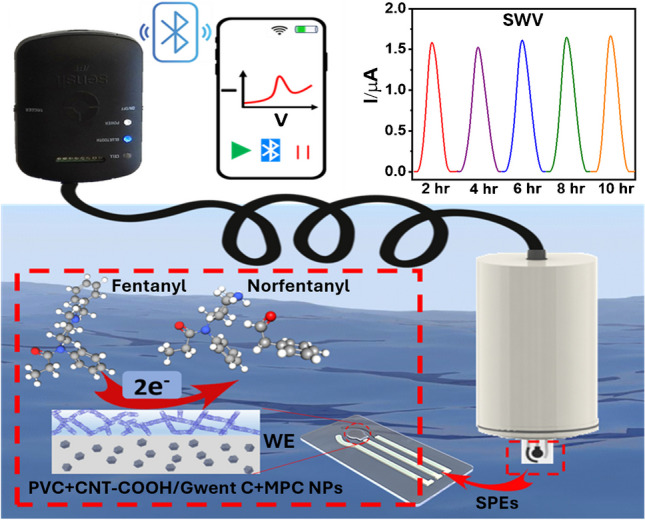

**Supplementary Information:**

The online version contains supplementary material available at 10.1007/s00604-024-06520-z.

## Introduction

Illicit drug manufacturing and trafficking globally endanger the lives of humanity. The USA has been particularly plagued by an alarming rise in opioid overdose deaths over the past two decades, from around 3.5 (2001) to 24 (2021) deaths per 100,000 [1], particularly owing to the persistent opioid trafficking across US borders [2], with a rising incidence of opioid lacing on counterfeit pills and recreational drugs [3]. Fentanyl (FT) and its analogs are acutely toxic synthetic opioid analgesics that have emerged as major contributors to overdose deaths in the USA [4, 5]. Ingestion or epidermal contact with FT residues results in rapid uptake into body fluids, leading to fatal outcomes due to rapid cessation of breathing and respiratory failure [6]. Such acute toxicity and widespread availability of FT have escalated the urgency for surveillance of FT trafficking and abuse [4, 7].

A prominent outcome of the COVID-19 pandemic has been the advancement of community wastewater-based epidemiology (WBE) toward faster and more accurate mapping of the source and spread of malicious water-borne threats [8]. Similarly, a scalable and cost-effective strategy for community mapping of the opioid crisis is by directly and remotely monitoring the water systems [9, 10]. Illicit FT-related activities are highly likely to contaminate proximate water systems, thus opening the opportunity for monitoring community opioid exposure to facilitate prompt alerts and precise legal enforcement [9]. Concerted efforts have accordingly been made to develop portable sensors for remote/on-site detection of trace FT contamination [11]. Field-deployable devices have thus been deployed, including mass spectrometer (MS) variants [12], liquid chromatography-mass spectrometer (LC–MS) variants [13], ion mobility spectrometers (IMS) [14], surface-enhanced Raman scattering (SERS) spectrometers [3, 15], electrochemical-SERS (EC-SERS) instruments [16], optical fiber sensors [17], colorimetric assay kits [18], or lateral flow immunoassay strips [19]. However, these approaches suffer from high costs, inadaptability, lengthy analytical procedures, and require trained professionals. In contrast, miniaturized electrochemical sensors [11, 20, 21] have emerged as rugged and low-cost alternatives for real-time, remote/on-site, and user-friendly operation. Notably, portable and wearable form factors of electrochemical impedance spectroscopic (EIS) [22] and voltammetric [6, 23, 24] FT sensors have exhibited an attractive combination of rapid multiphasic sensing, high selectivity, extended operational stability, and nanomolar sensitivity. As demonstrated in our current and previous studies, owing to the unmatched combined advantages of ultralow sub-micromolar limits of detection (LOD) and limits of quantification (LOQ), wide linear ranges from few nanomolar to hundreds of micromolar concentrations, response times of a few seconds, reagentless measurements without sample pretreatment, low sensor costs (< $1), high sensor operational stabilities of several hours, and high sensor storage stabilities at room temperature over several weeks, voltammetric FT sensors offer attractive performance characteristics for rugged and robust remote/on-site monitoring of trace FT contamination in uncontrolled field environments [6, 23, 24]. However, realizing the direct, reliable, and extended in situ operation of electrochemical sensors in real water systems can be challenging, owing to fluid convection, gradual sensor fouling, and/or unpredictable pH changes. Hence, despite considerable needs and advances in handheld/wearable electrochemical sensing systems for the remote/on-site field testing of illicit substances, no studies have been reported on direct and extended real-time FT profiling in remote underwater locations [15, 25].

**Fig. 1:**
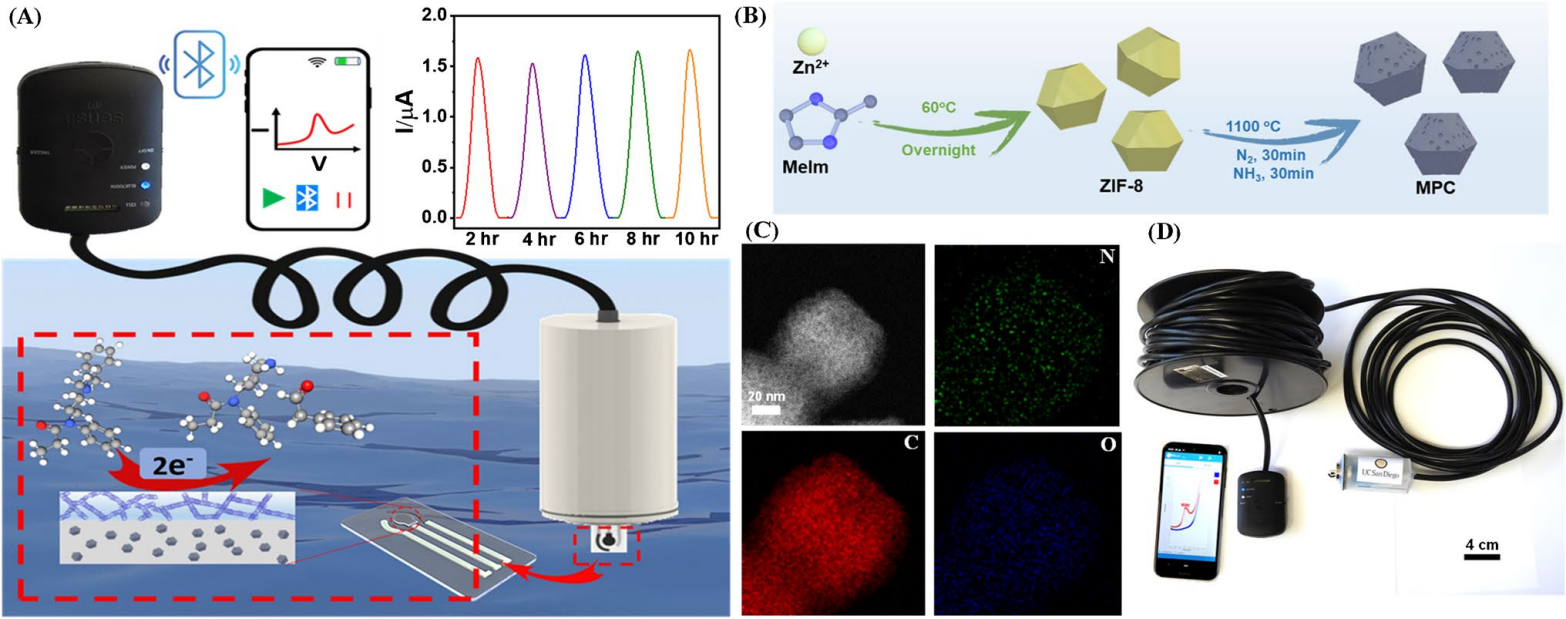
(**A**) Schematic of the remote submersible probe design and corresponding voltammetric FT sensor, along with FT detection principle and a 10-h-long FT sensing ability in domestic wastewater (WW). (**B**) Schematic illustration of the synthetic protocol for ZIF-8-derived N-doped mesoporous carbon (MPC) nanoparticles (NPs). (**C**) Dark field (DF)-STEM image of MPC NPs and corresponding C (red), N (green), and O (blue) elemental EDS mapping. (**D**) Real image of the remote opioid sensing probe for long-distance and extended submersible monitoring of FT, along with BLE-enabled wireless data acquisition

Herein, we demonstrate the pioneering development of a submersible opioid sensing probe that offers highly sensitive and stable remote underwater FT sensing at varying depths over extended durations (Fig. 1). Remote electrochemical sensors have been shown useful for continuous in situ monitoring of toxic metals and explosive residues [26]. To the best of our knowledge, the present probe represents the first demonstration of an electrochemical device for continuous in-situ environmental monitoring of opioid drugs. The new submersible voltammetric opioid probe was assembled by integrating a screen-printed voltammetric sensor chip with a 3D-printed waterproof housing unit. By systematically optimizing the electrode transducer and capping layer compositions, we have obtained remarkable sensor operational stability over several hours in domestic wastewater and untreated river water. The screen-printed carbon electrode (SPCE) transducer comprises zeolitic imidazolate framework-8 (ZIF-8)-derived N-doped mesoporous carbon (MPC) nanoparticles (NPs) blended in a conductive graphitic ink. The incorporation of MPC NPs serves to enhance the FT accumulation and direct electrocatalytic oxidation of FT [27, 28]. Additionally, we developed a new protective mixed-matrix membrane (MMM) formulation as the working electrode (WE) capping layer, which comprises a mixture of polyvinyl chloride (PVC) and carboxyl-functionalized multi-walled carbon nanotubes (CNT-COOH). Here, the lipophilic and insulating PVC helps to maximize the FT selectivity and operational stability [6], while the semiconductive CNT-COOH can serve as a supplemental electrocatalyst (aiding the MPC-based electrocatalytic transducer) for direct FT electrooxidation [29, 30]. Additionally, the hydrophilic carboxyl groups of CNT-COOH apparently helped mitigate the irreversible electrode passivation due to the progressive accumulation of the aromatic FT oxidation products [31], thus supporting PVC toward enhancing the sensor operational stability. Achieving such fouling resistance led to high sensor operational stability of over 10 h in domestic wastewater samples and over 4 h in untreated river water samples. The optimized FT sensor chip was finally integrated with a homemade submersible sensing probe, comprising a three-electrode electronic connector with a 30.5-m-long extension cable, to realize extended remote opioid monitoring across variable depths and distances. The submerged FT sensor chip could thus be operated using an external portable potentiostat (wirelessly interfaced via Bluetooth to a nearby smartphone) outside the water, which enabled rapid, real-time, and extended FT analysis using square wave voltammetry (SWV) (Fig. 1A). Overall, the developed submersible FT sensing probe displays distinct advantages of miniaturized in-situ electrochemical sensing systems toward remote community mapping and tracking of dynamic opioid contamination, for enhancing the global efforts of mitigating the opioid crisis.

## Experimental

The synthesis protocols for ZIF-8 MOF NPs and MPC NPs and the fabrication protocols for the voltammetric FT sensor and the remote opioid sensing probe have been explained in detail within the Supplementary information.

### Materials and reagents

Ag/AgCl ink (E2414, Ercon, Inc., MA, USA) and Gwent carbon ink (SunChemical, C2030519P4, Gwent Electronic Materials Ltd., UK) were used as obtained. Multi-walled carbon nanotubes (MWCNT, *Ø* = 10–20 nm, 10–30 µm length, > 99% purity) and carboxyl-functionalized multi-walled carbon nanotubes (MWCNT-COOH, *Ø* = 10–20 nm, 10–30 µm length, > 95% purity) were purchased from Cheap Tubes Inc. (Grafton, VT, USA; Richmond, CA, USA). Fentanyl (1 mg/mL, supplied in methanol, FT), heroin (1 mg/mL, supplied in acetonitrile, HN), and morphine (1 mg/mL, supplied in methanol, MN), urea, caffeine, acetaminophen, polyvinyl chloride (PVC), glacial acetic acid, toluene, hydrochloric acid (HCl), sodium hydroxide (NaOH), sodium chloride (NaCl), sodium carbonate (Na_2_CO_3_), glacial acetic acid, tetrahydrofuran (THF), zinc nitrate (Zn(NO_3_)_2_ 6H_2_O), 2-methylimidazole (C_4_H_6_N_2_), and methanol were purchased from Sigma–Aldrich (St. Louis, MO, USA) and used as received. Tetrahydrofuran (THF) was from Fisher Scientific (Hampton, NH, USA). Ultrapure water (resistivity ≥ 18.2 MΩ cm^−1^) was obtained from a Milli-Q system. The stencil patterns were designed via the AutoCAD 2021 software (Autodesk, San Rafael, CA, USA) and then ordered for fabrication on stainless steel, through-hole, 12″ × 12″ framed stencils of 125-µm thickness at Metal Etch Services (San Marcos, CA, USA). Transparent flexible PET sheets (thickness: 134 µm) were procured from McMaster-Carr (Robbinsville, NJ, USA). The Thermo Scientific Orion Star A211 benchtop pH meter (Catalog # STARA2110) was purchased from Thermo Fisher Scientific (Waltham, MA, USA).

### Instrumentation

Voltammetric FT sensing experiments were conducted by running SWV from *E*_begin_ =  − 0.4 V to *E*_end_ = 1.2 V (*E*_step_ = 4 mV, amplitude = 50 mV, frequency = 25 Hz), using a screen-printed miniaturized three-electrode electrochemical system [comprising a reference electrode (RE), working electrode (WE), and counter electrode (CE)] operated in various unbuffered aqueous samples. All voltammetric measurements were performed by inserting the screen-printed Ag/AgCl contact pads of the voltammetric FT sensor into the three-electrode connector of the PalmSens Sensit BT potentiostat (dimensions: 7.5 × 5.5 × 2.3 cm) that was wirelessly controlled via the PStouch software (version 2.8) on a nearby Bluetooth-enabled Android smartphone. Experiments on the FT sensors were performed using a 2.5-mL analytical aliquot of either 0.1 M phosphate buffer solution (PBS, pH 7.0) or other real water samples, including domestic wastewater (WW) and San Diego River water (SDRW). The working electrode of the screen-printed FT sensor was fully submerged in the liquid aliquot and secured using alligator clips and anti-static tape. The same electrochemical parameters were employed for the remote SWV operation of the voltammetric FT sensing probe, with the probe tip fully submerged in the aqueous sample matrix before SWV was performed.

## Results and discussions

Developing remote submersible probes for extended in situ monitoring requires proper attention to key issues, including reversibility, long-term stability, sensitivity, selectivity, and changes in natural conditions. ZIF-8-derived N-doped mesoporous carbon (MPC) nanoparticles (NPs) were used as electrode surface modifiers for imparting a highly sensitive FT response [27, 28]. The synthesis of N-doped MPC NPs is illustrated in Fig. 1B. First, the ZIF-8 precursor is synthesized by constructing tetrahedrally coordinated zinc ions linked by N-group in organic imidazole units. The typical process involves rapid mixing equimolar amounts of Zn(NO_3_)_2_·6H_2_O and 2-methylimidazole (MeIm) in a sealed round-bottom flask with methanol. The flask is then shaken and subsequently transferred into a 60 °C oven overnight. The precipitated white ZIF-8 precursors are collected after full filtration and washing. The MPC nanoparticles (NPs) are further obtained from a pyrolytic process of the above-collected ZIF-8 and treated at 1100 °C under an N_2_ atmosphere for 30 min, following a 30 min of NH_3_ gas treatment. The morphology and structure of ZIF-8-derived N-doped MPC were characterized by scanning transmission electron microscopy (STEM) and are shown in Supplementary Fig. S1. A polyhedral structure of MPC NPs with a mean NP size of around 80 nm is observed. The observed disordered graphitic layer structures around the MPC provide abundant defects and structural porosity. Such features will enable more doping of N species, which, along with existing structural defects, can boost electrocatalytic activity [32]. Besides, no NPs were observed over the carbon matrix during the bright field (BF)-STEM characterization, which means the involved Zn is evaporated during the pyrolysis process and a subsequent acid-washing step [33]. Energy dispersive X-ray spectroscopy (EDS) mappings were conducted to identify the rich N moieties (green) in the carbon matrix (Fig. 1C). The designed MPC is then employed by mixing it with the Gwent carbon ink to fabricate screen-printed electrodes for use as the voltammetric sensing probe after modification with a protective MMM, comprising an insulating PVC matrix and CNT-COOH as semiconductive fillers. A 3D-printed waterproofing unit, a portable Bluetooth-enabled potentiostat, and a customizable extension cable are integrated with the voltammetric sensing probe for rapid and extended remote monitoring of opioids upon submersion in community water systems (Fig. 1D). Figure 1A shows the performance of the voltammetric sensing platform over a 10-h-long remote FT monitoring in domestic wastewater (WW) spiked with 10 µM of FT. The background-subtracted SWV results show that a stable FT oxidation current signal intensity is obtained after every 2 h (one scan every 30 min), with only a small 5.18% current intensity variation between the first depicted scan (at 2 h) and the final measurement after 10 h. These data confirm that the new sensing platform holds good promise for extended FT monitoring in real water samples and significantly mitigates the potential decay of the FT oxidation current signal caused by organic electrode foulants and by FT oxidation product-induced electrode passivation.

**Fig. 2:**
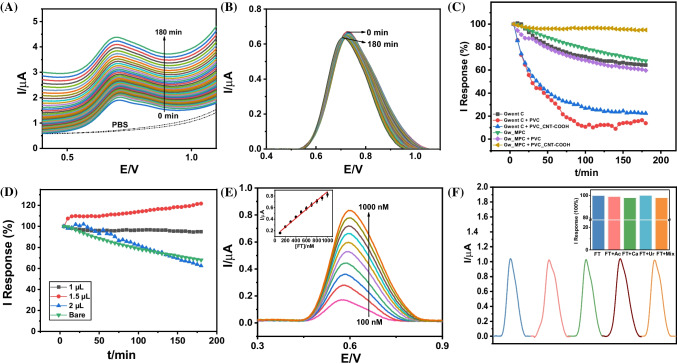
Electrochemical optimization of the sensor composition and characterization of the screen-printed voltammetric FT sensor chip in 2.5 mL of PBS (0.1 M, pH 7.0) spiked with FT. (**A**) Raw square wave voltammetric (SWV) data obtained for 10-µM FT oxidation from 0 to 180 min. (**B**) SWV baseline-subtracted data (moving average baseline, number of sweeps = 2, window size = 2000) corresponding to the raw SWV data obtained for FT oxidation from 0 to 180 min. (**C**) Normalized FT oxidation peak current response profiles (versus their original responses at *t* = 5 min) corresponding to the SWV moving average baseline-subtracted data obtained over 180-min at 5-min intervals, and comparison between the different investigated sensor compositions-Gwent C (black), Gwent C + PVC (red), Gwent C + PVC_CNT-COOH (blue), Gw_MPC (green), Gw_MPC + PVC (violet), and Gw_MPC + PVC_CNT-COOH (dark yellow). (**D**) Optimization of the PVC_CNT-COOH MMM thickness from 1 to 2 µL of drop cast volume on the Gw_MPC transducer. (**E**) Sensor response over the 0.1–1 µM FT range (100 nM additions), based on the SWV moving average baseline-subtracted FT oxidation peak current responses (*n* = 3), along with the corresponding calibration plot (inset). (**F**) Comparative moving average baseline-subtracted SWV response data obtained for the respective oxidation of 10-µM FT, 50-µM Ac, 50-µM Ca, 50-µM Ur, a mixture of 50-µM Ac, 50-µM Ca, and 50-µM Ur, and a mixture of 10-µM FT, 50-µM Ac, 50-µM Ca, and 50-µM Ur. Inset presents the comparative normalized response data (versus that of FT alone) obtained for the respective oxidation of FT, FT and Ac, FT and Ca, FT and Ur, and a mixture of FT, Ac, Ca, and Ur

Figure 2A depicts the raw SWV data for FT oxidation (10-µM FT spiked in PBS). Over an extended 3-h period. The SWV FT oxidation peak current depicted a remarkable response stability that is evident from the corresponding moving average baseline-subtracted peak current data in Fig. 2B. Supplementary Fig. S2 presents the reproducibility of the normalized current response profile from 5 to 180 min (versus FT response at *t* = 5 min) across three identically fabricated FT sensor chips, corresponding to the baseline-subtracted SWV data presented in Fig. 2B. The FT peak oxidation current in Fig. 2B exhibited a minor overall temporal decrease (versus original response at *t* = 0 min) between 102.2% (*t* = 5 min) and 95.0% (*t* = 180 min), with a maximum standard deviation (SD) of 10.71% at *t* = 65 min (*n* = 3 identically fabricated FT sensors) (Supplementary Fig. S2). Figure 2C illustrates the optimization study of the FT sensor composition based on (a) comparison of different transducer layers – Gwent carbon ink (Gwent C) versus 2.5 wt.% MPC-loaded Gwent C (Gw_MPC) – and (b) comparison of different polymeric capping layers (i.e., anti-passivation membranes) – 1 µL PVC versus 1 µL PVC_CNT-COOH. Supplementary Fig. S3 presents the comparative temporal profiles of SWV peak oxidation current obtained across the different FT sensor compositions over 3 h (one SWV forward scan every 5 min up to 180 min), toward oxidation of 10-µM FT (prepared in 2.5 mL of 0.1 M PBS, pH 7.0). It can be inferred from the comparison that incorporating MPC in a Gwent C ink-based printed transducer significantly improves the intensity of the FT response by around 30%. Hence, the elevated FT current response suggests that MPC significantly contributes to both the FT accumulation through the highly porous structure of MPC and to the electrocatalysis of FT due to the existence of nitrogen-rich defects (Fig. 1B). Next, addition of insulating PVC (i.e., Gw_MPC + PVC, violet) as a capping layer on Gw_MPC significantly reduces the FT oxidation peak current (by around 45%) but greatly improves the temporal response stability. The role of PVC as a lipophilic FT accumulation layer and an anti-interference barrier for less lipophilic coexisting interferents is also established in our previous work [6]. However, the addition of CNT-COOH to the PVC capping layer (i.e., Gw_MPC + PVC_CNT-COOH, dark yellow) relatively improves the FT oxidation peak current (around 25%, versus only PVC) due to the p-type semiconductive properties of CNT-COOH while further improving the temporal response stability. Moreover, the unique anti-passivation advantage of incorporating CNT-COOH in the PVC-based capping layer can be attributed to the hydrophilic carboxyl groups on the acid-treated walls of the MWCNTs that retard the progressive norfentanyl accumulation and related sensor passivation [31], which otherwise follows via strong π-π stacking interactions on the aromatic Gw_MPC-based electrode transducer [23, 34]. To establish the superiority of the selected sensor composition (i.e., Gw_MPC + 1 µL PVC_CNT-COOH) for sensing FT in PBS (0.1 M, pH 7.0), we systematically optimized the drop-casting volume of the PVC_CNT-COOH capping layer (from 0 to 2 µL) to optimize the signal stability and minimize the overall current deviation of the FT response over 180 min (Fig. 2D). It can be distinctly observed that the Gw_MPC + 1 µL PVC_CNT-COOH sensor composition exhibits optimal temporal response stability across the investigated 180-min time interval, with only a minor response decline of around 5% across the entire duration of 5 to 180 min. Supplementary Fig. S4 presents the raw SWV FT peak oxidation current response profiles corresponding to the comparative data presented in Fig. 2D. We observed that although the 2-µL capping layer exhibited faster and higher sensor passivation over 180 min (37.5% response decline) versus both 1 µL (5.1% response decline) and 1.5 µL (22% response increase), it exhibited lower overall passivation than the uncapped bare Gw_MPC electrode (i.e*.*, 0 µL, 32% response decline over 180 min) up to *t* = 130 min. We thus optimized the capping layer thickness as 1 µL to minimize the electrode passivation and thereby extend the FT SWV oxidation current response stability [6, 34]. Supplementary Fig. S5 depicts the moving average baseline-subtracted peak current data for the optimized Gw_MPC + 1 µL PVC_CNT-COOH sensor composition toward the SWV oxidation of 5–50-µM FT spiked in 2.5 mL of 0.1 M PBS (pH 7.0). The FT oxidation current response exhibited a linear increase (Adj. *R*^2^ = 0.995) with increasing FT concentration (inset calibration plot), yielding a detection sensitivity of 48 nA/µM [FT] across the entire investigated micromolar concentration range. Figure 2E depicts the moving average baseline-subtracted peak current data for the optimized FT sensor toward the SWV oxidation of 0.1–1-µM FT spiked in 2.5 mL of 0.1 M PBS (pH 7.0). The FT sensor exhibited a desirable linear response (*R*^2^ = 0.988, *n* = 3) across the investigated sub-micromolar FT concentration range, with a theoretical limit of detection (LOD) of around 29.5 nM (i.e., 9.9 µgL^−1^, as calculated from the inset calibration plot, as 3 × slope/SD of *y*-intercept). This LOD value of around 30 nM FT is lower than the ~ 60 nM lethal threshold of FT in human blood [6]. Similarly, the limit of quantification (LOQ) of FT was determined to be around 98 nM FT (calculated from the inset calibration plot as 10 × slope/SD of *y*-intercept), which matches closely with the concentration of the lowest recorded SWV oxidation peak current for a 100-nM [FT] addition. Notably, the incorporation of ZIF-derived, N-doped MPC NPs in Gwent C ink was essential to obtain the required nanomolar FT oxidation current sensitivity, as well as the resulting sensitivity of the norfentanyl oxidation peak (as obtained at E_pa_ =  − 0.15 V and shown later in Fig. 4D, G and Supplementary Figs. S11 and S12) for FT discrimination from other opioids upon successive anodic SWV scans [6, 23]. The attractive sensitivity of the voltammetric FT sensor chip suggests its strong potential for detection of trace FT contamination (below the lethal human threshold) in untreated real water samples. Supplementary Table S1 presents the analytical performance comparison between the current voltammetric FT sensor chip and existing portable/wearable prototypes of voltammetric FT sensors. Notably, the PVC_CNT-COOH-modified Gwent C_MPC SPCE sensor chip exhibits a superior combination of nanomolar FT sensitivity (LOD and LOQ < 100 nM [FT]), low response time (10 s), wide linear range (0.1–50-µM [FT]), and unprecedented operational stability (94.90% normalized response after 36 scans (3 h) in 10-µM FT in PBS), thus establishing viability of the current voltammetric FT sensor chip for real-time, direct, and extended in-situ monitoring of trace FT levels in untreated community water systems.

Figure 2F demonstrates the selectivity of the FT sensor (using 10 µM FT) in the presence of common aqueous interferents and FT cutting agents, including 50 µM each of caffeine (Ca), acetaminophen (Ac), and urea (Ur). The baseline-subtracted FT oxidation peak currents were 1.042 µA for FT alone, 1.024 µA for FT + Ac, 1.030 µA for FT + Ca, 1.041 µA for FT + Ur, and 1.022 µA for FT + Ac + Ca + Ur, respectively. The obtained raw SWV data corresponding to the above interferents study has been depicted in Supplementary Fig. S6. The inset of Fig. 2F presents the normalized response profiles (versus the oxidation peak current obtained for FT alone), corresponding to the respective baseline-subtracted oxidation peak current responses. Caffeine and acetaminophen are commonly used as FT cutting agents, with urea also being highly abundant in wastewater systems as a human and industrial effluent [15, 25]. These compounds are all potential interferences with the FT sensor’s electrochemical performance. Yet, the FT sensor chip displayed a remarkable selective response with signal retention of 98.27% for FT + Ac, 98.85% for FT + Ca, 99.90% for FT + Ur, and 98.08% for FT + Ac + Ca + Ur, respectively. Such remarkable selectivity toward FT detection can be attributed to the combined advantages of distinct oxidation peak potentials obtained for different electroactive molecules using SWV-based electroanalysis, along with the high FT selectivity of the lipophilic PVC_CNT-COOH MMM placed on the voltammetric transducer. Overall, the results of the selectivity study suggest considerable promise for applying the FT sensor chip to remote field analysis of untreated community water samples.

**Fig. 3:**
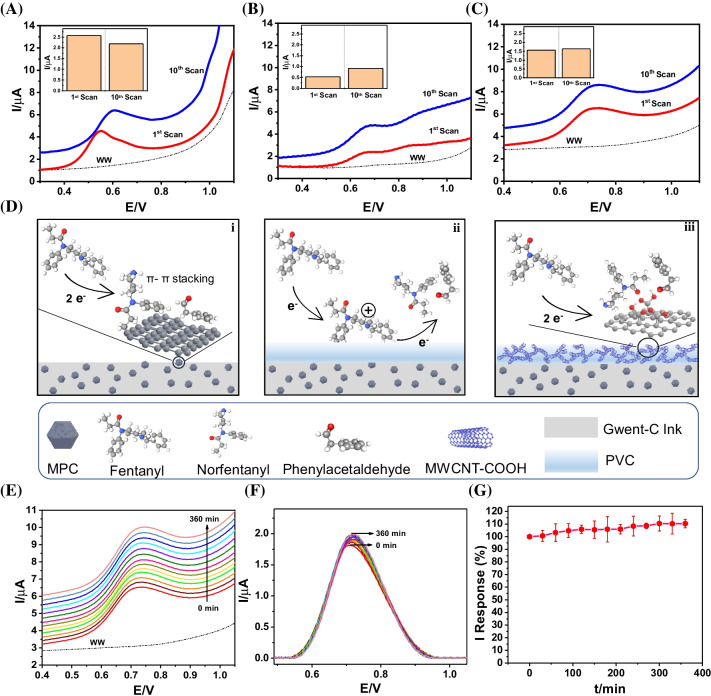
Extended wastewater (WW) FT monitoring and the anti-fouling sensor coating strategy. Comparison of the 1st and 10th raw SWVs scans at (**A**) bare Gw_MPC sensor, (**B**) Gw_MPC + 1.5-µL PVC sensor, and (**C**) Gw-MPC + 1.5-µL PVC_CNT-COOH sensor toward sensing 10-µM FT spiked in domestic WW. Consecutive SWV scans are taken at a 30-min interval. Inset graphs represent the corresponding baseline-subtracted current signal intensity for each sensor composition. (**D**) Schematic illustration of the proposed electrochemical interactions between FT and various sensor compositions, including (i) bare Gw_MPC sensor, (ii) Gw_MPC + 1.5 µL PVC sensor, and (iii) Gw_MPC + 1.5 µL PVC_CNT-COOH sensor. (**E**) Raw SWV data obtained for oxidation of 10-µM FT spiked in domestic WW, intermittently analyzed from 0 to 360 min using the optimized sensor composition of Gw-MPC + 1.5 µL PVC_CNT-COOH. (**F**) SWV baseline-subtracted data (moving average baseline, number of sweeps = 2, window size = 2000) corresponding to the raw SWV data obtained for 10-µM FT oxidation from 0 to 360 min. (**G**) Normalized 10-µM FT oxidation peak current response profiles (versus their original responses at *t* = 0 min), corresponding to the SWV moving average baseline-subtracted data (*n* = 3) obtained over 360 min at 30-min intervals

Figure 3A demonstrates the SWV sensing performance of the bare Gw_MPC sensor toward extended and intermittent FT sensing in domestic WW. The highly porous structure of MPC NPs affords a large specific surface area of graphitic carbon. This enables a high accumulation capacity of FT on the sensor surface via π-π stacking interaction between FT and MPC [6, 23]. Moreover, the abundant electrocatalytically active nitrogen defects enable a high degree of catalytic SWV oxidation of the accumulated FT, thereby enhancing the FT sensitivity [28]. Although the sensor’s strong FT accumulation affinity and electrocatalytic activity results in high 1st scan SWV signal intensity (2.572 µA), the large amount of oxidized FT also drastically increases electrode passivation due to surface adsorption of norfentanyl and phenylacetaldehyde [6, 23]. The electrode passivation by these oxidation products inhibits future FT from directly accumulating onto active MPC regions of the sensor surface, thus diminishing the electrocatalytic activity of MPC (Fig. 3D(i)). This causes a drastic decrease in the SWV oxidation signal obtained, as reflected in the 0.383 µA decrease between the 1st and 10th scans in the bare Gwent-C-MPC sensor, amounting to a significant 14.89% intensity decrease at a nearly identical FT concentration in just 10 consecutive scans (at 30-min intervals). To address this electrode passivation issue, a protective capping layer was introduced onto the sensor surface. Early studies reported that the lipophilic characteristics of PVC can effectively improve FT accumulation and improve sensor selectivity [6]. However, the raw SWV data in Fig. 3B demonstrate that if the capping layer is solely PVC, the signal intensity drastically decreases to the sub-1 µA level for the 10 µM FT sample, thus making it impractical to monitor sub-micromolar FT concentrations that are likely to exist in real wastewater samples. We deduced that this sharp decrease in sensitivity is due to the insulating nature of the PVC membrane, which diminishes the electron transfer rate between FT and the underlying voltammetric transducer (Fig. 3D(ii)), thus causing the FT oxidation signal to exhibit two oxidation peaks [21] in the SWV raw data of Fig. 3B. The twin peak occurrence (around 0.68 V and 0.85 V) precludes the reliable quantification of FT based on a single oxidation peak intensity. To increase the electron transfer rate, we thus incorporated semiconductive CNT-COOH fillers into the insulating PVC matrix to obtain a protective MMM. The effective nanocomposite self-assembly (within the dispersion medium of THF), comprising the semiconductive carboxylated multi-walled carbon nanotubes (CNT-COOH) incorporated within the PVC dielectric matrix, is facilitated by a high spatial density of Van der Waals interactions at the interfaces of CNT-COOH and PVC, which are promoted by the carboxyl groups on CNT-COOH [35]. The inclusion of CNT-COOH increases the conductivity of the PVC membrane [34], thus eliminating the occurrence of 2 FT oxidation peaks (Fig. 3C). We selected carboxyl group-functionalized MWCNTs, instead of pristine MWCNTs, because CNT-COOH is more dispersible in the PVC solution in THF (Supplementary Fig. S7), which results in a more uniform deposition of the protective MMM on the FT sensor compared to pristine MWCNTs. The improved dispersion of CNT-COOH in PVC, versus that of pristine CNTs (Fig. S7), is also in strong agreement with the results of the above-cited work on similar PVC-CNT and PVC-CNT-COOH -based anti-fouling membranes [35]. As depicted in the schematic illustration in Fig. 3D(iii), CNT-COOH is less likely to form aggregates within the PVC matrix as compared to its pristine MWCNTs counterpart, which agrees with the unviable MMM obtained with pristine MWCNTs. The relative stability of the two types of MWCNT dispersions can also be visually confirmed by their corresponding optical images taken over 60 min (pristine MWCNTs (left) vs. CNT-COOH (right)) (Supplementary Fig. S7). In addition, a uniform density of surface carboxyl groups on the CNT-COOH reduces the likelihood of π-π stacked FT oxidation products, thus helping mitigate temporal electrode passivation [31] (Fig. 3D(iii)). Figure 3C shows that the current signal intensity of the 10th consecutive SWV scan after 300 min (at 30-min intervals) is relatively stable compared to the initial SWV scan. The inset shows a current intensity difference of merely 0.078 mA after 300 min, amounting to only 5.01% variation compared to the initial SWV scan. It is important to point out that the bare Gw_MPC SPCE transducer (Fig. 3A) displays a significantly higher FT oxidation current for the 1st forward square wave voltammetric (SWV) scan versus both the PVC-coated SPCE (Fig. 3B) as well as the optimized PVC_CNT-COOH-coated SPCE (Fig. 3C). Moreover, this bare SPCE continues to have a higher FT oxidation current after the 10th forward SWV scan (30-min interval between consecutive SWV scans), spanning a total intermittent analytical period of 5 h for all three versions of the voltammetric FT sensor. Hence, it can be safely concluded that the electroactive surface area of the bare Gw_MPC SPCE transducer is significantly higher than its anti-fouling membrane-coated sensor counterparts, which is owed to the insulating nature of PVC within the membrane. However, the novelty of this work is the optimization of a unique anti-fouling PVC_CNT-COOH MMM that affords unprecedented operational stability of 4–10 h (> 95% normalized FT oxidation response) during extended intermittent FT monitoring (one forward SWV scan every 30 min) in diverse untreated water samples ranging from domestic WW to San Diego River water (SDRW). Using the optimized sensor composition for domestic WW analysis (*i.e.,* Gw-MPC + 1.5 µL PVC_CNT-COOH), Fig. 3E depicts the raw SWV data for FT oxidation in WW spiked with 10 µM FT. The SWV FT oxidation peak current displays a good temporal response stability over 6 h of intermittent operation, which is evident from the corresponding moving average baseline-subtracted peak current data in Fig. 3F. Finally, the current response normalized with respect to the initial current intensity also shows that after 360 min (i.e., after 12 scans at 30-min intervals) of sensor deployment, the current signal intensity only varied 10.36% (*n* = 3) compared to the initial scan response (Fig. 3G). Such high sensor operational stability in benchtop WW solutions indicated viability for integration of the voltammetric FT sensor chip on a remote submersible probe for extended in-situ field monitoring of opioid contamination at varying depths.

**Fig. 4:**
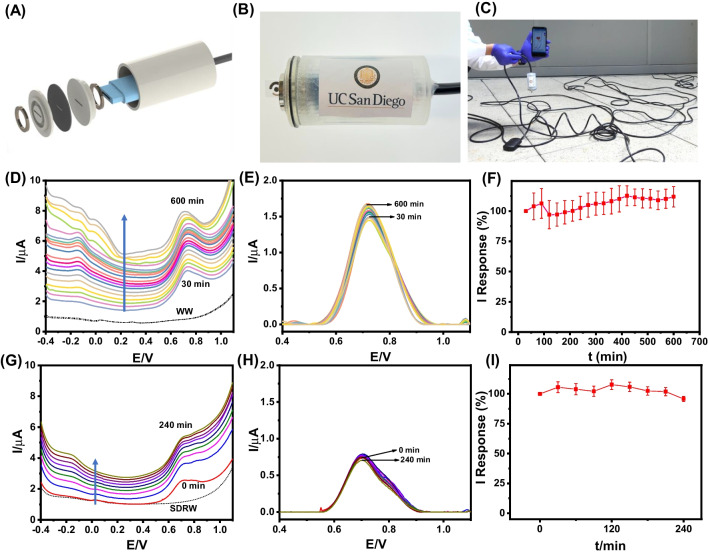
In situ FT monitoring. Extended remote monitoring of FT contamination (10-µM spiked FT) in 2.5 mL of untreated real water samples using the screen-printed voltammetric FT sensor chip (2.5 wt.% MPC in Gwent C and 1.5/2 µL (domestic wastewater, WW/San Diego River water, SDRW) of PVC_CNT-COOH), and the in-house constructed submersible FT sensing probe. (**A**) Fabrication schematic for the assembly of the submersible remote FT sensing probe. (**B**) Real image of the constructed submersible FT sensing probe comprising the chemically modified screen-printed voltammetric FT sensor chip interfaced with a three-electrode electronic connector and wired integration of the electronic connector with a 30.5-m electrical extension cable to enable large-distance and extended submersible operation of the remote FT sensor. (**C**) Real image depicting the large-distance operation of the remote FT sensing probe, including the employed electrochemical data acquisition apparatus comprising a computer-interfaced PalmSens Sensit BT portable potentiostat, which is wired to the extension cable of the submerged voltammetric opioid sensing system. (**D**) Raw SWV FT oxidation peak current responses obtained from *t* = 30–600 min (waiting time interval of 30 min) in a domestic WW sample (pH 8.54). (**E**) Absolute FT oxidation peak current responses obtained by baseline subtraction (moving average baseline, number of sweeps = 2, window size = 2000) of the raw SWV peak current data obtained from *t* = 30–600 min in WW. (**F**) Normalized FT oxidation peak current response profile (versus the original response at *t* = 30 min) corresponding to the baseline-subtracted SWV peak current data (*n* = 3) obtained from *t* = 30–600 min. (**G**) Raw SWV FT oxidation peak current responses obtained over 4 h, from *t* = 0–240 min (at 30-min intervals), in an untreated SDRW sample (pH 7.8). (**H**) Absolute FT oxidation peak current responses obtained by baseline subtraction (moving average baseline, number of sweeps = 2, window size = 2000) of the raw SWV peak current data obtained from *t* = 0–240 min in SDRW. (**I**) Normalized FT oxidation peak current response profile (versus the original response at *t* = 0 min) corresponding to the baseline-subtracted SWV peak current data (*n* = 3) obtained from *t* = 0–240 min

Upon previously verifying the reliable benchtop analytical performance of the FT sensor chip in WW samples, subsequent efforts focused on implementing the envisioned field opioid sensing application by integrating the new voltammetric FT sensor chip on the tip of the submersible probe of a remote large-distance electrochemical sensing system. As shown in the 3D schematic in Fig. 4A, a submersible electrochemical sensing probe was constructed in-house using a 3D-printed rigid polymeric enclosure, two magnetic spacers, a three-electrode electronic connector, and the voltammetric FT sensor chip. The assembled probe was then wired to a customizable extension cable to enable long-distance underwater opioid monitoring (up to a depth of tens of meters), ultimately enabling the extended duration monitoring of field FT contamination in domestic and natural aqueous environments. Figure 4B depicts a real image of the constructed remote FT sensing probe, and Fig. 4C depicts a real image of the employed experimental setup for large-distance remote submersible operation of the developed electrochemical FT sensing system (by using a 30.5-m-l extension cable). The large-distance and submerged operation of the remote FT sensing probe in WW was realized by its integration with an electrochemical data acquisition apparatus comprising a Bluetooth-enabled PalmSens Sensit BT portable potentiostat, which was wired to the extension cable of the submerged voltammetric opioid sensing probe. This sensing system enabled wireless real-time data acquisition on the user’s smartphone.

Figure 4D–F presents the 10-h remote electrochemical response stability study of the assembled remote FT sensing probe submerged in domestic WW (pH 8.54) spiked with 10-µM FT. Figure 4D depicts the raw square wave voltammetric (SWV) data obtained for the oxidation of 10-µM FT spiked in 2.5 mL of WW, with a 30-min waiting time interval between consecutive anodic SWV scans. The WW sample was prepared by mixing 100 mg urea, 10 mg potassium chloride, and 10 mg sodium phosphate (to simulate the constituents of human liquid excretions) [36], and the resultant solution was obtained when a human subject washed their hands with 1.5-mL Safeguard liquid hand soap and 1-L tap water from the university restroom. A stable FT response was observed across the entire duration of 10 h (with negligible temporal shift of the FT oxidation peak potential (E_pa_) ≈ + 0.72 V) by employing the previously optimized FT sensor composition (2.5 wt.% MPC in Gwent C + 1.5 µL PVC_CNT-COOH). Notably, high FT response selectivity has been verified by the absence of unwanted peaks in the baseline voltammogram of FT-free WW (Fig. 4D), suggesting no interference from other dissolved contents of the WW sample like liquid hand soap, urea, KCl, and Na_3_PO_4_. Additionally, the SWV oxidation peak of the norfentanyl oxidation product of FT (E_pa_ =  − 0.15 V) validates the high fidelity of opioid discrimination by employing SWV analysis for extended monitoring of untreated and complex aqueous media [6, 23]. Such FT selectivity indicates good promise for future field FT testing in other community water systems (like sewage water).

To further investigate the capability of our voltammetric opioid sensor toward simultaneous detection and discrimination of distinct opioids in separate and mixture samples, we have conducted opioid discrimination studies using a more informative electroanalytical technique – cyclic square wave voltammetry (CSWV) based on previous studies [6, 23] (Supplementary Fig. S11 and Fig. S12**)**. The obtained results in stirred 0.1 M PBS (pH 7.0) validate that CSWV yields unique electrochemical signatures for distinct opioids, including FT, MN, and HN, in their separate and mixture samples. In summary, FT exhibits an irreversible oxidation to norfentanyl (NT) at around + 0.70 V, followed by a partially reversible redox couple around − 0.25 V corresponding to surface-adsorbed NT [36]. This partially reversible redox couple of NT exhibits sensitive and nearly linear peak current increases (especially for the NT reduction peak, Adj. *R*^2^ = 0.973) with repeated CSWV scans for a single FT addition (Supplementary Fig. S12B), which indicates the increasing surface adsorption of NT with repeated FT oxidation scans for the same FT addition. Importantly, Supplementary Fig. S12A validates the linear (Adj. *R*^2^ = 0.997) and sensitive (62 nA/µM [FT]) FT oxidation response, while Supplementary Fig. S12B validates retention of the unique electrochemical signature of FT obtained using CSWV in 10 mL of 0.1 M PBS (pH 7.0), which are in strong agreement with the electrochemical signature (Supplementary Fig. S11A), linearity (Adj. *R*^2^ = 0.995), and sensitivity (48 nA/µM [FT]) (Supplementary Fig. S5) obtained originally in 2.5 mL of 0.1 M PBS (pH 7.0). In contrast with the electrochemical signature of FT, HN exhibits only one irreversible oxidation peak at around + 0.65 V (Supplementary Fig. S11B). Furthermore, MN exhibits two irreversible oxidation peaks at around + 0.18 V and + 0.65 V, along with two irreversible reduction peaks at around + 0.25 V and − 0.2 V (Supplementary Fig. S11C). Lastly, the mixture of equimolar FT + HN + MN exhibits the unique irreversible MN oxidation peak around + 0.18 V, followed by a large irreversible oxidation peak around + 0.70 V corresponding to the combined oxidation of FT, HN, and MN (Supplementary Fig. S11D). A larger peak current versus that of exclusive NT reduction (Supplementary Fig. S11A) was observed for the reduction peak obtained around − 0.2 V, which indicates the joint contribution of NT and MN. These results are in strong agreement with the voltammetric opioid sensing results presented in our earlier studies [6, 23]. Hence, CSWV has now been demonstrated on our voltammetric opioid sensor chip toward discrimination of distinct opioids in separate and mixture samples.

Figure 4E depicts the corresponding moving-average baseline-subtracted SWV peak current data (based on raw data presented in Fig. 4D) from *t* = 30–600 min. Remarkable response stability is observed across the entire duration of 10 h, versus only 70% response stability after 2 h in WW with a 10-min waiting time interval between consecutive SWV scans (Supplementary Fig. S8). This temporal SWV response behavior suggests that increasing the waiting-time interval plays a key role in mitigating the FT oxidation product-induced passivation of the aromatic carbon-based voltammetric FT transducer. Supplementary Fig. S9 presents the temporal FT response profile corresponding to the absolute SWV oxidation peak currents obtained across the investigated duration of 600 min (i.e., one SWV forward scan every 30 min up to 600 min). The absolute peak current displayed a gradual variation from 1.490 µA at 30 min to 1.549 µA after 1 h, 1.446 µA after 2 h, 1.529 µA after 4 h, 1.613 µA after 6 h, 1.646 µA after 8 h, and a stable response of 1.667 µA retained after 10 h. Such highly stable FT oxidation peak current response is attributed to the capping layer-induced diffusion limitations coupled with the minimal FT consumption over the investigated period of 10 h. Figure 4F presents the normalized FT oxidation peak current response profiles (normalized versus original response at *t* = 30 min), corresponding to the temporal peak current response profile presented in Supplementary Fig. S9. It can be observed that the FT sensor exhibits a gradual variation in SWV response from 103.91% normalized response at 1 h to 97.01% response after 2 h, 102.61% response after 4 h, 108.20% after 6 h, 110.44% after 8 h, and finally 111.85% response after 10 h. This amounts to a maximum normalized response deviation of only 11.85% (*n* = 3) over the entire 30–600-min period.

Upon verifying the high operational stability of the remote FT sensing probe submerged in domestic WW, we next investigated its operational stability in San Diego River water (SDRW). Figure 4G–I presents the analytical results obtained during a 4-h-long remote FT monitoring while the sensing probe submerged in untreated SDRW (pH 7.8) spiked with 10-µM FT. Figure 4G depicts the raw SWV data obtained for the oxidation of 10-µM FT spiked in 2.5 mL of SDRW, with a 30-min waiting time interval between consecutive anodic SWV scans. A stable FT oxidation response was observed across the entire duration of 4 h (with negligible temporal shift of the FT oxidation peak potential (E_pa_) =  + 0.70 V) by employing a 30-min waiting time interval with a newly optimized FT sensor composition (2.5 wt.% MPC in Gwent C + 2 µL PVC_CNT-COOH). The analytical results for the operational stability study conducted on the 1 µL (A-C) and 1.5 µL PVC_CNT-COOH (D-F) capping layers have been presented for comparison in Supplementary Fig. S10. In contrast with the previously optimized capping layer thickness for WW, an inferior operational stability was observed with only 1.5 µL of PVC_CNT-COOH (Supplementary Fig. S10D–F) versus that obtained with the newly optimized 2 µL of PVC_CNT-COOH (Fig. 4G–I). This can be attributed to the higher content of foulants expected in SDRW versus domestic WW, which inevitably leads to a faster rate of secondary electrode fouling (apart from FT oxidation product-induced electrode fouling) and apparently demands a higher thickness (2 µL in SDRW versus 1.5 µL in WW) of the protective PVC_CNT-COOH MMM. After optimization of the MMM thickness (2 µL), high FT response selectivity was verified by the absence of unwanted peaks in the baseline voltammogram of untreated SDRW (Fig. 4G), suggesting no redox interference from other dissolved contents of the SDRW sample like inorganic salts, heavy metals, and organic compounds. Additionally, the SWV oxidation peak of the norfentanyl oxidation product of FT (E_pa_ =  − 0.15 V) once again validates the high fidelity of opioid discrimination by employing SWV analysis for extended monitoring of untreated and complex aqueous media [6, 23]. Figure 4H depicts the corresponding moving average baseline-subtracted peak current data (based on raw SWV data in Fig. 4G) from *t* = 0–240 min. A remarkable temporal response stability was observed across the entire duration of 4 h. The absolute peak current displayed a gradual variation from 0.7362 µA at 0 min to 0.777 µA after 30 min, 0.765 µA after 1 h, 0.793 µA after 2 h, 0.754 µA after 3 h, and finally to 0.705 µA after 4 h. Such attractive temporal stability of the FT oxidation peak current response is attributed to the capping layer-induced diffusion limitations coupled with the minimal FT consumption over the investigated period of 4 h. It is important to clarify that the experiment was continued beyond 4 h, but the electrode fouling, unfortunately, became prominent (> 10% response decay) beyond this duration. Figure 4I presents the normalized FT oxidation peak current response profiles (normalized versus original response at *t* = 30 min) corresponding to the temporal peak current response profile presented in Fig. 4H. It can be observed that the FT sensor exhibits a gradual variation in SWV response from 105.52% normalized response at 30 min to 103.97% response after 1 h, 107.73% response after 2 h, 102.40% after 3 h, and finally 95.79% response after 4 h. This amounts to a maximum normalized response deviation of only 5.21% (*n* = 3) over the entire duration of *t* = 0–240 min.

Such high temporal response stability toward field monitoring of FT can be attributed to the systematic optimization of (a) wt.% of the MPC (2.5 wt.%) in the Gwent C-based FT transducer, (b) drop casting volume of the PVC_CNT-COOH (1.5 µL for WW and 2 µL for SDRW) capping layer, and (c) waiting time interval (30 min) between consecutive anodic SWV scans within the FT-contaminated aqueous solution. The above three sensor parameters altogether dictate the impact of FT oxidation product-induced sensor passivation (with the waiting interval playing a predominant role), which is the primary bottleneck to direct electrooxidative FT sensing over extended durations. It can also be inferred that the higher biofouling rates in domestic WW and SDRW necessitated a higher surface coverage of the PVC_CNT-COOH MMM (1.5 µL and 2 µL drop cast volume, respectively) for optimal operational stability versus that in PBS (1 µL drop cast volume). Notably, this work demonstrates the use of a protective MMM to prolong the operational stability of fouling-prone electrochemical opioid sensors, thus obviating the need for destructive electrode cleaning steps between consecutive opioid measurement scans [37]. Future efforts will focus on establishing a universally applicable composition of the protective MMM that eliminates the need to selectively optimize the capping layer thickness for a specific type of real water sample while ideally helping reduce the current waiting time (30 min) between consecutive FT measurement scans. Here, it is important to point out that the exact FT recovery percentages were not calculated for the analyzed untreated water samples (domestic wastewater and San Diego River water), considering our goal of establishing high operational stability (4–10 h with > 95% normalized response) of the voltammetric FT sensor chip. Our ongoing studies are focused on the rigorous calibration of the voltammetric FT sensor in diverse untreated water samples and the associated optimization of FT recovery percentages. Overall, the results of the extended real sample monitoring study suggest good promise for applying the voltammetric FT sensor chip to the remote field analysis of untreated community water samples.

Solution resistance plays a vital role in dictating the analytical performance of electrochemical sensors, as well as the mobility of protonated tertiary amine-containing opioids like fentanyl, morphine, and heroin [21]. Hence, considering the possibility of encountering opioids also in non-aqueous (i.e., dissolved in organic solvents) and/or solid phase (e.g., powders and dried residues) within the environment, we could then employ wearable/robotic electrochemical sensing platforms and partition opioids from a solid/non-aqueous phase to an electrochemically compatible gel electrolyte phase by interfacing a hydrogel disc with the voltammetric opioid sensor. This strategy can help realize on-site swipe, scan, and sense voltammetric analyses of multiphasic opioid samples [24].

## Conclusions

We have demonstrated that FT can be monitored continuously at large sample/instrument distances in untreated community water by coupling a judicious transducer surface modification technology with a submersible electrochemical sensor probe. The sensitivity of the remote voltammetric FT sensor has been uniquely enhanced using the incorporation of ZIF-8 MOF-derived MPC NPs within the screen-printed graphitic carbon ink. The abundant N-doping, high 3D porosity, and high specific surface area of MPC NPs enhanced their electrocatalytic activity and FT accumulation capacity, which enabled sensitive SWV FT detection down to 9.9 µgL^−1^ in PBS. Moreover, a major bottleneck related to phenolic compound-associated electrode fouling (here, oxidation products of FT) on graphitic carbon electrodes has been overcome using the integration of a PVC-CNT-COOH MMM on top of the MPC-based electrode transducer. Optimization of the MMM thickness yielded unprecedented operational stability of the FT sensor, ranging over 4–10 h in untreated and stirred community water samples, including river water and domestic wastewater. This electrode fouling resistance was attributed to the combination of a lipophilic anti-interference PVC barrier with semiconductive CNT-COOH fillers, where the hydrophilic carboxyl functionalities apparently played a vital role in mitigating the irreversible surface adsorption of aromatic FT oxidation products [31]. Overall, by enclosing the electronic interface of the FT sensor chip inside a 3D-printed waterproof housing unit, we could integrate a customizable extension cable wired to an external Bluetooth-enabled portable potentiostat. This unique approach for designing remote electrochemical opioid sensing probes demonstrates robust sensing performance toward meeting the urgent need for intermittent and extended underwater opioid monitoring at large distances and variable depths. Our ongoing efforts are focused on integrating a permselective size-exclusion membrane around the FT sensor to filter out macroscopic organic particulates present in community water. Additionally, we are trying to develop a universally applicable composition of the protective MMM that potentially eliminates the need to optimize the MMM thickness for a specific composition of real water samples. These advances are expected to enable the extended monitoring of opioids in more informative wastewater media, like sewage water, thus paving a viable path toward comprehensive WBE through wider and more accurate mapping of community opioid exposure.

### Supplementary Information

Below is the link to the electronic supplementary material.

Supplementary file1 (DOCX 4.28 MB)

## Data Availability

All supporting experimental details and data have been provided within the *Supplementary Information*.
