# Supplementary material for: Submersible voltammetric sensing probe for rapid and extended remote monitoring of opioids in community water systems
Source: Mikrochim Acta. 2024 Jul 12;191(8):463. doi: 10.1007/s00604-024-06520-z (PMC11245449; doi:10.1007/s00604-024-06520-z)
Supplement: Supplementary file 1 — Supplementary file1 (DOCX 4.28 MB) [file 604_2024_6520_MOESM1_ESM.docx]

**Supporting Information**

**Submersible Voltammetric Sensing Probe for Rapid and Extended Remote Monitoring of Opioids in Community Water Systems**

Jiachi Zhou,^+,a^ Shichao Ding,^+,a^ Samar S. Sandhu,^+,a^ An-Yi Chang,^a^ Anubhap Taechamahaphan,^a^ Shipra Gudekar,^a^ Joseph Wang^a,^*

*^a^Department of Nanoengineering, University of California, San Diego, La Jolla, California 92093, United States*

*^+^These authors contributed equally to this manuscript*

**Corresponding author: J. Wang, ORCID: 0000-0002-4921-9674*

*Email address:* [*josephwang@ucsd.edu*](mailto:josephwang@ucsd.edu)

*Full address: Department of Nanoengineering, University of California, San Diego, La Jolla, California 92093, United States*

**Experimental**

1. **ZIF-8 MOF nanoparticles (NPs) Synthesis Protocol**

The synthetic procedure was adopted from previous works[1, 2], that was adapted from the original work of Ding *et. al.*[3, 4]_._ Zinc Nitrate Hexahydrate (3.39g, 0.014 mol) was added to an oven dried, griffin beaker filled with 150 ml methanol and covered with parafilm. In a separate griffin beaker filled with 150ml methanol, 2-Methylimidazole (3.94, 0.048 mol) was added and then covered with parafilm. The two beakers are both sonicated until all solutes are dissolved, both followed with a 2 min nitrogen (N_2_) purge. The two solutions are then both poured to a round bottom flask. The flask is sealed with rubber seal immediately after. The flask is then sonicated for 5 min and then shaked vigorously for 2 min. The mixture in the round bottom flask is then transferred into a pre-heated oven at 60 degrees Celsius for 24 h. After a significant quantity of white solids (ZIF-8 MOF precursor**)** has fully precipitated at the bottom of the flask, the flask is transferred out of the oven and allowed to cool to room temperature. Afterwards, the heterogeneous mixture containing insoluble ZIF-8 MOF precursor is transferred into a centrifuge tube. Centrifugation at 6000 rpm was performed to collect the precursor. The precursor is then vacuum filtered using a 1-µm pore sized PTFE microfiber membrane. The collected solid is washed multiple times with methanol and then water, then with ethanol to ensure remaining reagents are removed. The white ZIF-8 MOF precursor is then collected, and oven dried for 6 hours at 60 °C. Afterwards, the fully dried ZIF-8 MOF is ground with mortar and pestle until fine powder-like consistency is attained. This powder was then collected in a glass vial for further MPC NPs synthesis procedure.

1. **Mesoporous Carbon (MPC) NPs Synthesis Protocol**

The final ZIF-8 derived, nitrogen-doped MPC NPs were obtained from the pyrolytic process of ZIF-8 MOF precursors at 1100°C under argon and nitrogen atmosphere for 30 min, respectively. Here, the furnace is heated from room temperature to 1100°C at a rate of 5°C /min. The collected nitrogen-doped MPC NPs are then acid washed using 3M HCl at 60 °C for 6 h to remove any residual metal impurities and then the MPC NPs are dried in the oven at 60 °C for 6 h.

1. **Fabrication of the Voltammetric Fentanyl Sensor Chip**

SPEs were fabricated on flexible PET substrates by using a semiautomatic MPM–SPM screen printer machine (Speedline Technologies, Franklin, MA) and stainless steel, through-hole, 12″ × 12″ framed stencils of 125 µm thickness. The corresponding stencil designs were developed using the AutoCAD 2021 software. The screen printing and heat curing procedure of the sensor electrodes on the flexible PET substrate was based on the previously reported procedure[5] and comprised the following steps: first, Ag/AgCl conductive ink (Ercon E2414) was used to print the contact pads, the conductive collector traces and the reference electrode (RE), and were cured at 95 °C for 20 min. Next, the working electrode (WE) and counter electrode (CE) were printed with either Ercon carbon ink (E3449), or customized N-MPC containing carbon ink that was prepared by vortex mixing Ercon carbon ink and 2.5 w.t % N-MPC. The printed carbon ink layers were cured at 80 °C for 10 min. Finally, a SEBS formulation (MD 1648, 1:5 w/w in Toluene) was used to print the final sensor electrical insulation layer, and was cured at 60 °C for 10 min. The SPEs were stored at room temperature until further use.

1. **Working Electrode (WE) Modification Protocol of the Voltammetric Fentanyl Sensor Chip**

The modification step of the screen-printed WE involves the drop casting of the PVC or PVC-CNT solution for the formation of the anti-fouling membrane. For PVC only solutions, 0.2 mg of PVC is added to 10 mL of THF and then sonicated until dissolved. For PVC-CNT or PVC-CNT-COOH solutions, 0.4 mg of CNT or CNT-COOH is added to 10 mL of 20 µg/mL PVC solution, respectively. The solution is then sonicated until the CNT or CNT-COOH is well dispersed in the solution, respectively. For the membrane drop casting, a 0.5 µL aliquot of the investigated membrane solution is drop cast evenly on the WE, uniformly covering all the WE surface. After the solvent is fully evaporated, this drop casting process is repeated until the desired total drop cast solution volume is attained.

1. **Detailed Fabrication Procedure of the Remote Voltammetric Opioid Sensing Probe**

The lid, sensor socket, and casing of the remote sensing probe are all 3D printed using a FormLabs 3+ printer. The adhesive is then applied to the sensor socket to attach the aluminum strips, spaced apart with respect to the distance of the sensor electrodes, serving as the electrical connection part. A 3mm thick 3-line phone cable is routed through the probe casing and first soldered to the back end of the connection part of the sensor socket, then sealed with silicone glue (Gorilla Silicone Sealant) to ensure the casing is waterproof. The other end of the 3-line phone cable is then connected to the PalmSens Sensit BT potentiostat. The lid of the sensing probe is glued on with a strong circular magnet and a circular rubber pad matching the diameter of the casing, followed by aligning a circular magnet on the sensor socket to ensure that the lip can be properly sealed and aligned with the socket when closed. The FT sensor chip is then inserted into the sensor socket through both the laser-cut opening on the lid and then the rubber.

**Supporting Figures**


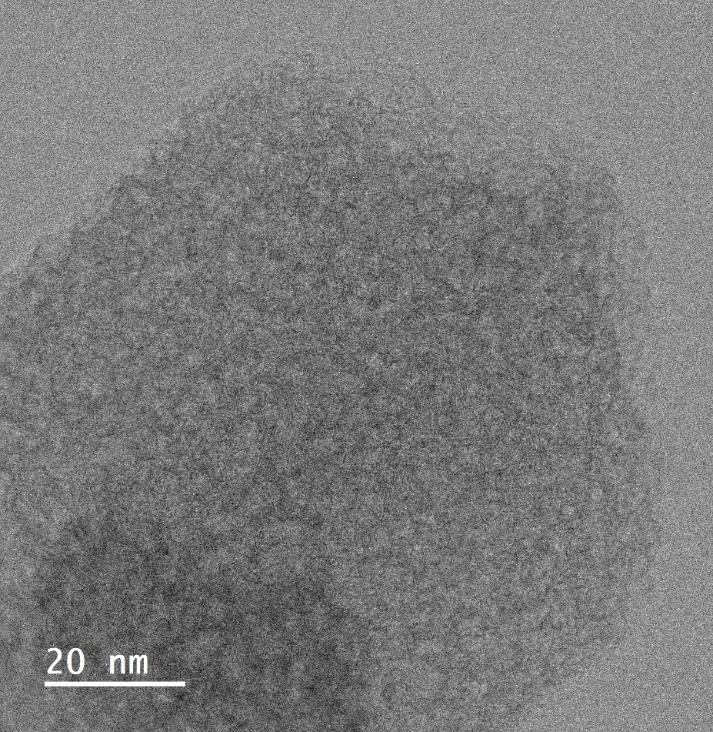


**Fig. S1. Bright field (BF)-STEM characterization of ZIF-8 MPC NPs.** Scanning Transmission Electron Microscopy characterization of the ZIF-8 derived mesoporous carbon nanoparticles.

b)

a)

**Fig. S2. Reproducibility study for identically fabricated FT sensors in 2.5 mL of stirred 0.1 M aqueous phosphate buffer solution (PBS), pH 7.0.** Reproducibility of the normalized current response profiles from 5 to 180 min (versus FT response at t = 5 min) across the remaining two identically fabricated (n=3) FT sensor chips. a) Sensor number 2, b) Sensor number 3. (Sensor number 1 shown in **Fig 2B**)

**Fig. S3. Comparative study for optimizing sensor composition toward extended FT response stability in PBS.** Remaining comparative temporal profiles of SWV peak oxidation current obtained across all the investigated FT sensor compositions over 3 h (one SWV forward scan every 5 min up to 180 min), towards oxidation of 10 µM FT (spiked in 2.5 mL of stirred 0.1 M PBS, pH 7.0). Investigated compositions include a) Gwent-C ink; b) Gwent-C ink, 1 µL PVC membrane; c) Gwent-C ink, 1 µL PVC+CNT-COOH mixed-matrix membrane (MMM); d) Gwent-C+2.5 wt.% MPC ink; e) Gwent-C+2.5 wt.% MPC ink, 1 µL PVC membrane.

**Fig. S4. Comparative study for optimizing sensor MMM thickness toward extended FT response stability in PBS.** Raw SWV FT (10 µM) peak oxidation current response profiles (in 2.5 mL of stirred 0.1 M PBS (pH 7.0)) of the remaining investigated sensor MMM thicknesses corresponding to different drop cast volumes of- a) 1.5 µL, and b) 2.0 µL, using a mixture of 20 µg/mL PVC and 40 µg/mL CNT-COOH dispersed in THF.

**Fig. S5. Calibration study of the optimized FT sensor composition in PBS.** The figure depicts the moving average baseline-subtracted peak current data for the optimized Gw_MPC+1 µL PVC_CNT-COOH sensor composition, towards the SWV oxidation of 5-50 µM FT spiked in 2.5 mL of stirred 0.1 M PBS (pH 7.0). The inset presents the linear calibration plot of the obtained SWV oxidation peak currents with increasing 5 µM FT additions.

**Fig. S6. Specificity data of the optimized FT sensor in PBS.** Raw SWV data of 10 µM FT sensing in 2.5 mL of stirred 0.1 M PBS (pH 7.0), containing 50 µM of either Acetaminophen, Urea, Caffeine, or 50 µM each of all three chemical interferents.


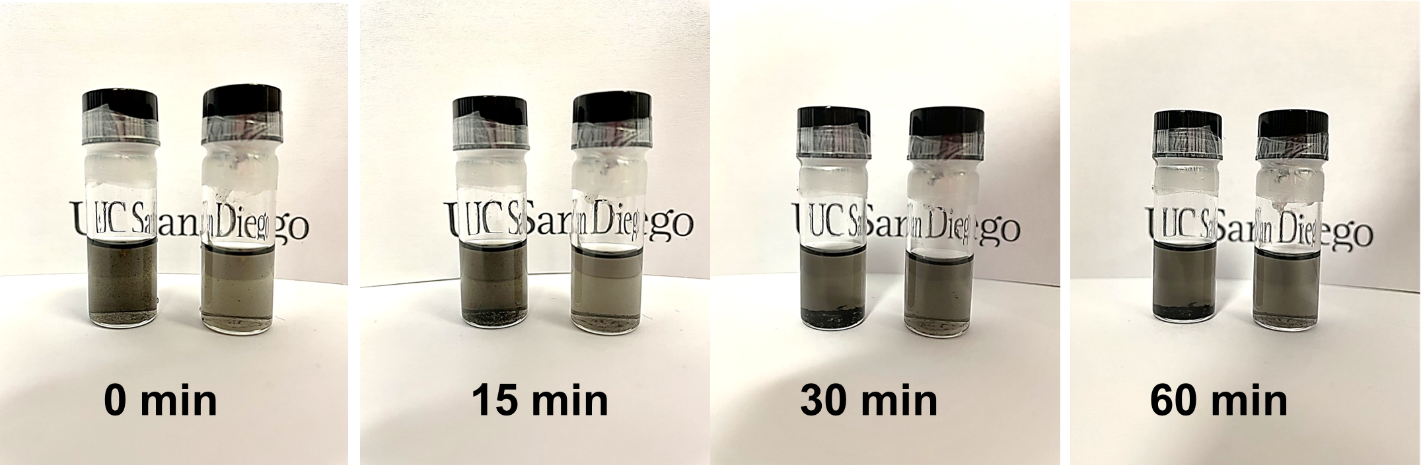


**Fig. S7. Dispersion stability study of pristine MWCNT and MWCNT-COOH dispersions in THF.** Visual images of the two types of MWCNTs (pristine vs. functionalized) dispersed in THF (MWCNT left, MWCNT-COOH right) after bath ultrasonication for 20 min. The pictures were taken immediately, 15min, 30min, and 60min after removal from the ultrasonication bath, respectively.

**Fig. S8. FT Accumulation Time Optimization Study for FT Sensor in domestic wastewater, WW**. 10-min waiting time interval between consecutive SWV FT (10 µM) oxidation scans for a period of 2 hours. a) Raw SWV data of the FT sensor, b) moving average baseline-subtracted peak current data.

**Fig. S9. Temporal FT response stability in domestic wastewater (WW).** This profile corresponds to the absolute SWV FT (10 µM) oxidation peak currents obtained across the investigated duration of 600 min (*i.e.*, one SWV forward scan every 30 min up to 600 min) in domestic WW. a) Raw SWV data of the FT sensor, b) moving average baseline-subtracted peak current data.

**(e)**

**(d)**

**(c)**

**(b)**

**(a)**

**(f)**

**Fig. S10. Protective MMM layer optimization study for extended FT response stability in San Diego River water (SDRW).** This operational stability study corresponds to optimization of the PVC_CNTCOOH MMM thickness from (a-c) 1 µL, to (d-f) 1.5 µL on the FT sensor submerged in SDRW. Results from the SWV FT (10 µM) oxidation peak currents obtained across the investigated duration of 240 min (*i.e.*, one SWV forward scan every 30 min up to 240 min) have been displayed.

**Fig. S11. Opioid discrimination study using cyclic square wave voltammetry (CSWV) for simultaneous detection of distinct opioids in PBS.** CSWV responses obtained for 5-15 µM additions of (A) FT, (B) Heroin (HN), (C) Morphine (MN), and (D) Equimolar mixture of FT+HN+MN, in 2.5 mL of stirred 0.1 M PBS pH 7.0, using the optimal 1 µL PVC_CNTCOOH MMM coating thickness on the Gwent C_MPC voltammetric SPCE transducer.

**Fig. S12. Effect of higher solution volume on the analytical performance of the voltammetric FT sensor in PBS.** (A) CSWV responses obtained for 5-15 µM additions of FT in 10 mL of stirred 0.1 M PBS pH 7.0, using the optimal 1 µL PVC_CNTCOOH MMM coating thickness on the Gwent C_MPC voltammetric SPCE transducer. Inset presents the corresponding linear calibration plot. (B) Repeated CSWV scans conducted after the 15 µM FT addition in 10 mL of stirred 0.1 M PBS, pH 7.0. Inset presents the corresponding linear calibration plots corresponding to the progressively increasing redox peak currents of the FT oxidation product, norfentanyl.

**Table S1. Comparison of the Analytical Performance of the PVC_CNT-COOH-modified Gwent-C_MPC SPCE-based Voltammetric FT Sensor with Previously Reported Portable/Wearable Voltammetric FT Sensors*^a^***

*^a^*IL = Room-temperature ionic liquid, Gwent C = Screen-printable Gwent carbon ink, Ercon C = Screen-printable Ercon carbon ink, SPCE = Screen printed carbon electrode, PVC = Polyvinyl chloride, CNT-COOH = Carboxylated multi-walled carbon nanotubes, PEI = Polyethyleneimine, MPC = ZIF-8 MOF-derived N-doped mesoporous carbon nanoparticles, fCNFs = Carboxyl-functionalized carbon nanofibers, PBS = Phosphate buffer solution, CSWV = Cyclic square-wave voltammetry, SWV = Square-wave voltammetry, DPV = Differential pulse voltammetry, LOD = Limit of detection, LOQ = Limit of quantification.

**Supporting References**

1. Xiao M, Zhu J, Ma L, et al (2018) Microporous Framework Induced Synthesis of Single-Atom Dispersed Fe-N-C Acidic ORR Catalyst and Its in Situ Reduced Fe-N4 Active Site Identification Revealed by X-ray Absorption Spectroscopy. ACS Catal 8:2824–2832. https://doi.org/10.1021/acscatal.8b00138

2. Li JC, Xiao F, Zhong H, et al (2019) Secondary-Atom-Assisted Synthesis of Single Iron Atoms Anchored on N-Doped Carbon Nanowires for Oxygen Reduction Reaction. ACS Catal 9:5929–5934. https://doi.org/10.1021/acscatal.9b00869

3. Ding S, Barr JA, Lyu Z, et al (2023) Effect of Phosphorus Modulation in Iron Single-Atom Catalysts for Peroxidase Mimicking. Adv Mater. https://doi.org/10.1002/adma.202209633

4. Ding S, Barr JA, Shi Q, et al (2022) Engineering Atomic Single Metal–FeN 4 Cl Sites with Enhanced Oxygen-Reduction Activity for High-Performance Proton Exchange Membrane Fuel Cells. ACS Nano 16:15165–15174. https://doi.org/10.1021/acsnano.2c06459

5. Chang A, Sandhu SS, Fernando PUAI, et al (2023) Electrochemically Induced Conformational Change of Di‐Boronic Acid‐Functionalized Ferrocene for Direct Solid‐State Monitoring of Aqueous Fluoride Ions. Adv Funct Mater 33:1–18. https://doi.org/10.1002/adfm.202303968
